# Sequential Bilateral Pneumothorax Following Acupuncture Presenting as Left Tension Pneumothorax: A Case Report

**DOI:** 10.7759/cureus.113708

**Published:** 2026-07-31

**Authors:** Sharmin Jahan Nitol, Aya Twabie, Ado Yusuf, Abbeygayle Worton, Mohammed Elashahab

**Affiliations:** 1 Acute Medicine, North Manchester General Hospital, Manchester University NHS Foundation Trust, Manchester, GBR

**Keywords:** acupuncture, bilateral pneumothorax, chest drain, iatrogenic pneumothorax, tension pneumothorax

## Abstract

Acupuncture is commonly used for musculoskeletal pain and is generally regarded as safe; however, pneumothorax remains one of its most important mechanical complications, particularly when needling is performed over thoracic and upper-back sites. We report the case of a 30-year-old man who developed immediate pleuritic chest pain, chest tightness, and dyspnoea after acupuncture to the upper back, bilateral trapezius muscles, and infrascapular regions. He delayed presentation until the following morning, when symptoms worsened. Examination demonstrated tachypnoea, reduced left-sided air entry, and hyperresonance. Chest radiography showed a large left tension pneumothorax with near-complete collapse of the left lung. A 20 Fr Seldinger chest drain was inserted under local anaesthesia with prompt improvement. Repeat imaging showed partial left re-expansion and a small right-sided pneumothorax. Digital monitoring with Thopaz (Medela AG, Baar, Switzerland) confirmed cessation of the air leak, and computed tomography (CT) of the thorax demonstrated complete left-sided resolution, a small residual right pneumothorax, and no structural lung abnormality. This case highlights delayed presentation despite immediate symptom onset, the possibility of bilateral pleural injury after bilateral thoracic needling, and the importance of repeat imaging when the initial pathology appears unilateral.

## Introduction

Acupuncture is a form of traditional complementary medicine originating from China and is now widely practised worldwide. It is used for several indications, including back pain, sports-related injury, myofascial pain, and chronic musculoskeletal syndromes. In the present case, acupuncture was performed to treat exercise-related upper-back pain. Although acupuncture is generally perceived as safe, serious complications are well described, including infection, haemorrhage, cardiac tamponade, and pneumothorax [1,2]. Among these, pneumothorax is one of the most clinically significant mechanical complications. Its reported incidence has been estimated at fewer than one per one million procedures; however, the true incidence is likely under-reported [1]. Diagnosis may be delayed because symptoms can initially be subtle or may not be immediately recognised, allowing potentially life-threatening deterioration [3,4].

Risk is determined by anatomy as much as technique. The upper trapezius, suprascapular, paraspinal, and infrascapular regions are particularly vulnerable because of the close proximity of the pleura to the skin and underlying musculature, particularly in lean individuals [3,4]. Needle length, angle, depth, and bilateral treatment during a single session all influence the risk of pleural injury. Pneumothorax may occur when thoracic acupuncture is performed with excessive depth or inappropriate needle direction and may not always be immediately recognised during the procedure.

Most published acupuncture-related pneumothoraces are unilateral and simple; bilateral pneumothorax and tension physiology are less common, but their rarity should not reassure clinicians [5,6]. Bilateral pleural injury is clinically important because a dominant pneumothorax may mask a smaller contralateral pneumothorax, potentially delaying diagnosis and influencing subsequent management. Recent reports continue to highlight this uncommon but potentially life-threatening complication. Kashiura et al. described bilateral pneumothorax following thoracic acupuncture, reinforcing the need to consider bilateral pleural injury even when the initial clinical or radiographic findings appear unilateral [7].

This report describes a case in which a dominant left-sided pneumothorax with evolving tension features masked a smaller contralateral pneumothorax that became apparent on repeat imaging, adding to the limited literature describing sequential recognition of bilateral pneumothoraces following acupuncture.

## Case presentation

A 30-year-old man presented to the emergency department with chest tightness, pleuritic chest pain, and dyspnoea. His symptoms began immediately following acupuncture for exercise-related upper-back pain. Acupuncture needles had been inserted across the upper back, including both trapezius muscles and the infrascapular regions. He had no significant past medical history and reported smoking one to two cigarettes per day.

He weighed 73 kg and was 179 cm tall, corresponding to a body mass index (BMI) of 22.8 kg/m². A history of recent COVID-19 infection was not documented and could not be confirmed retrospectively.

Despite the immediate onset of symptoms, he did not seek medical attention until the following morning because of persistent dyspnoea and pleuritic discomfort. The reason for delaying presentation and whether specific post-procedure safety netting advice had been provided could not be established retrospectively.

On presentation, he was tachycardic, with a heart rate of 130 beats/minute, and had a respiratory rate of 18 breaths/minute. He was otherwise haemodynamically stable, maintained satisfactory oxygenation without supplemental oxygen, and had no evidence of overt circulatory compromise. Chest examination demonstrated reduced air entry over the left hemithorax with hyperresonance on percussion, while breath sounds were preserved on the right. The remainder of the examination was unremarkable.

**Figure 1:**
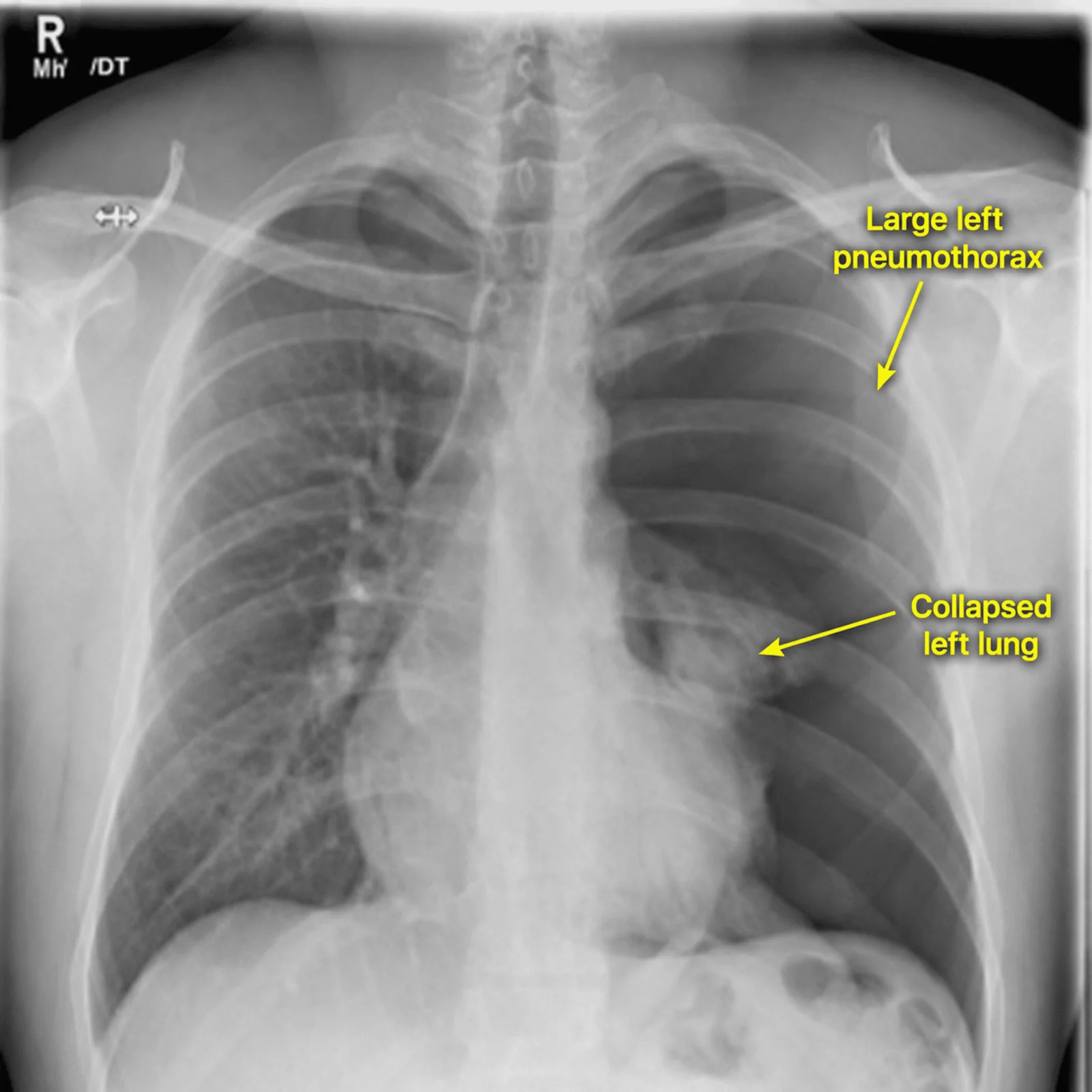
Initial chest radiograph: large left-sided pneumothorax with near-complete collapse of the left lung and subtle mediastinal shift, concerning for evolving tension physiology The annotations identify the visceral pleural line, extensive pleural air, collapsed left lung, and mediastinal displacement.

The initial chest radiograph demonstrated a large left-sided pneumothorax with near-complete collapse of the left lung and subtle mediastinal shift, raising concern for evolving tension physiology (Figure 1). Although the radiographic findings required urgent treatment, the patient remained haemodynamically stable, maintained satisfactory oxygenation, and had no evidence of obstructive shock. As definitive pleural drainage was immediately available, a 20 Fr Seldinger intercostal chest drain was inserted directly without preceding needle decompression.

The drain was inserted into the left pleural cavity through the fifth intercostal space at the mid-axillary line, resulting in immediate symptomatic improvement. Initial bubbling was observed, followed by cessation of bubbling while drain swinging continued.

**Figure 2:**
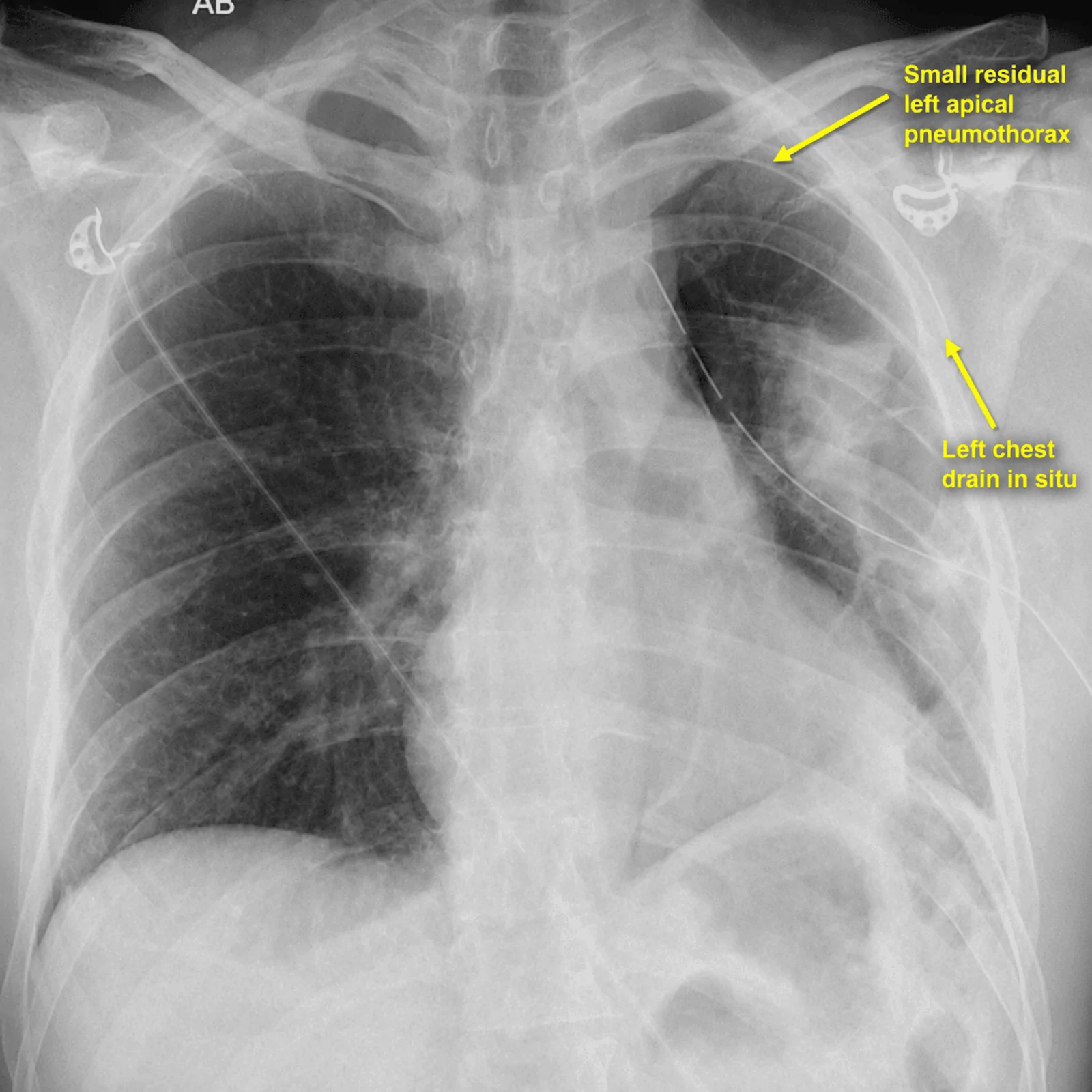
Post-drain chest radiograph: interval re-expansion of the left lung following insertion of the left intercostal chest drain, with a small residual left apical pneumothorax The annotations indicate the residual pleural air and chest-drain position.

**Figure 3:**
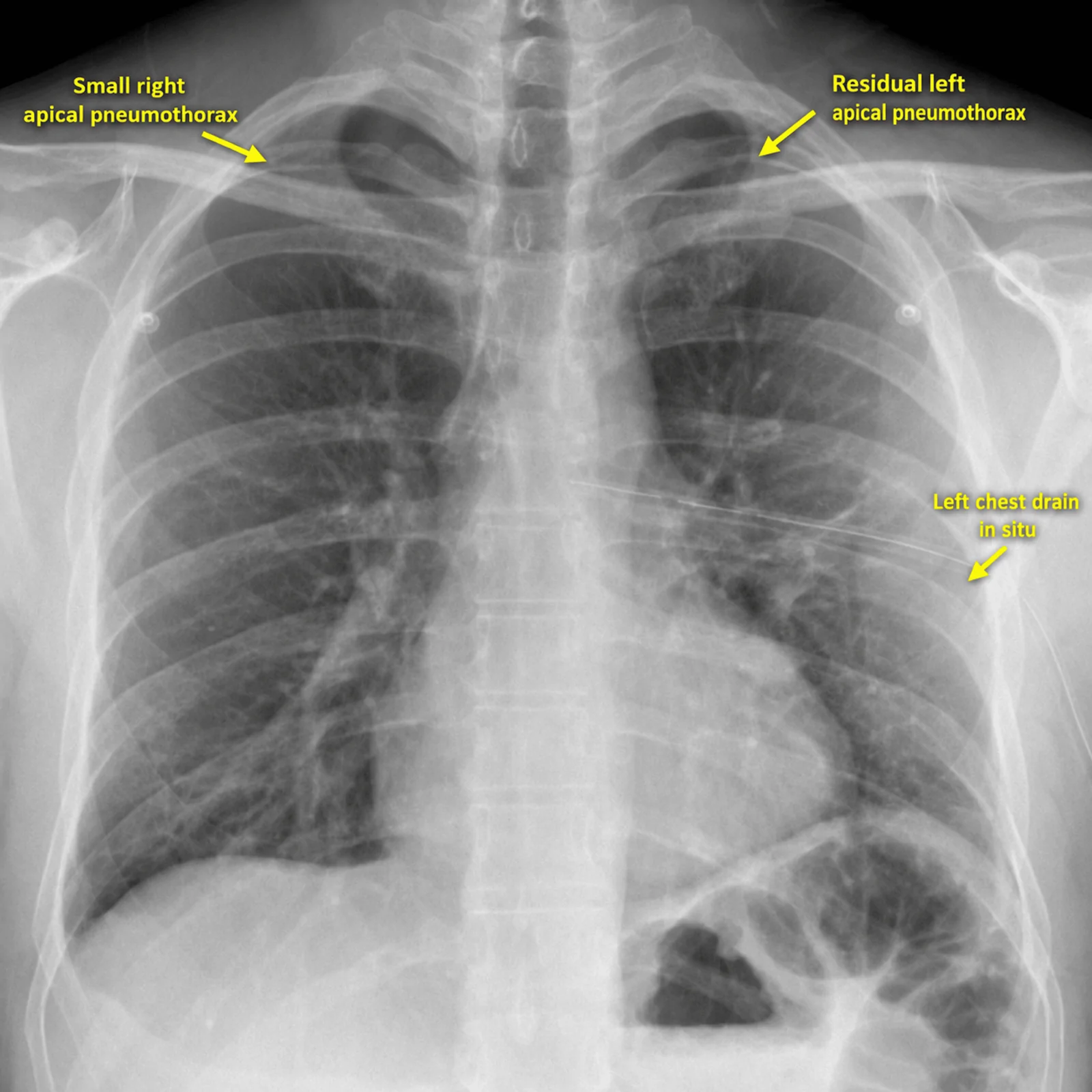
Separate follow-up chest radiograph: small right apical pneumothorax identified after treatment of the dominant left-sided pneumothorax The annotation identifies the right apical pleural line.

Repeat chest radiography demonstrated partial re-expansion of the left lung and identified a small right-sided apical pneumothorax that had not been appreciated on the initial radiograph (Figure 2 and Figure 3). A digital drainage system (Thopaz, Medela AG, Baar, Switzerland) was connected and demonstrated cessation of the air leak. Quantitative air-leak and intrapleural-pressure measurements were not available retrospectively.

**Figure 4:**
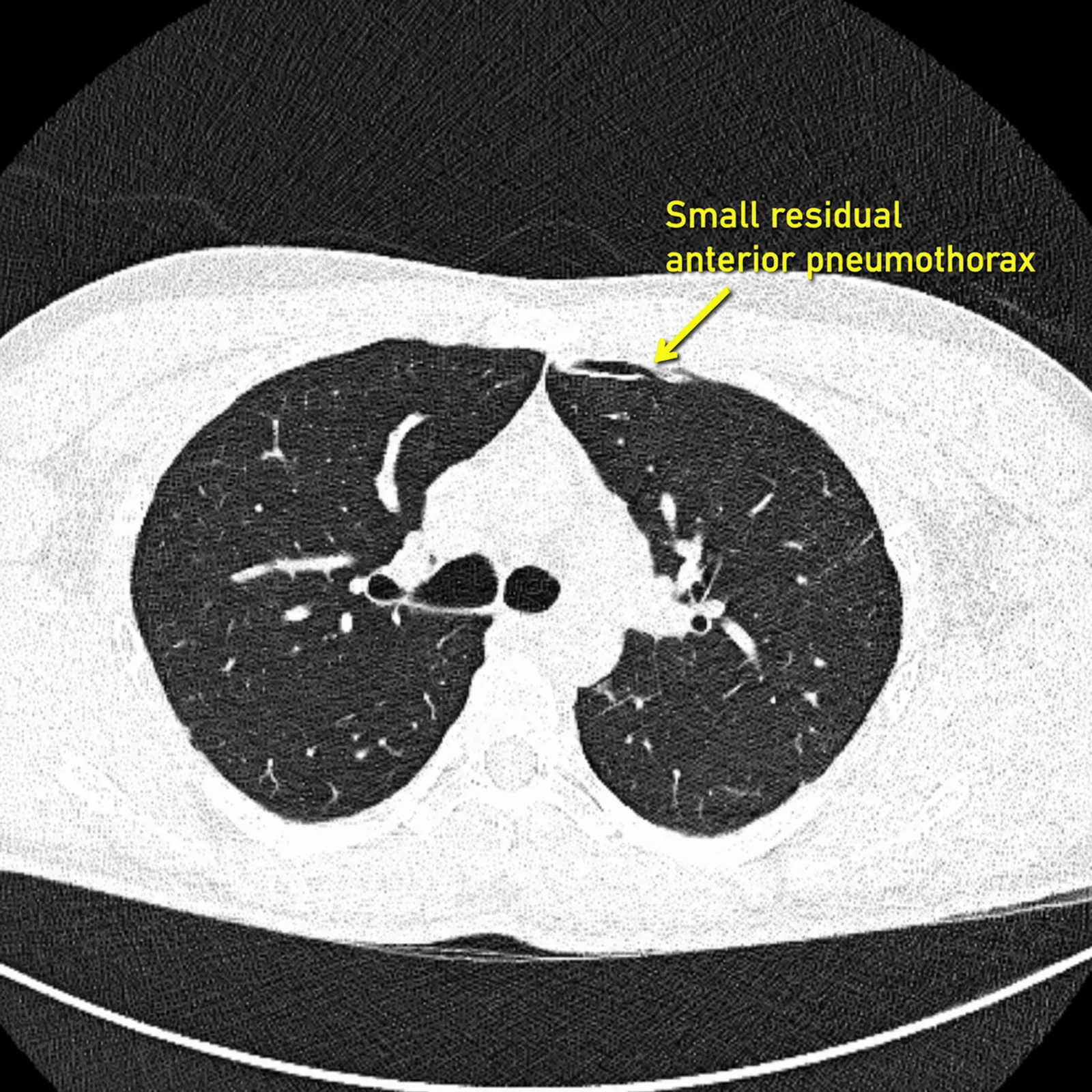
CT of the thorax: complete resolution of the left pneumothorax, a small residual right pneumothorax, and no significant structural lung abnormality The annotation identifies the residual right-sided pleural air. CT: computed tomography

Computed tomography (CT) of the thorax performed during the same admission confirmed complete resolution of the left pneumothorax, a small residual right pneumothorax, and no significant structural lung abnormality (Figure 4). Following cardiothoracic review, the chest drain was removed on the third day, and conservative management was advised for the small right-sided apical pneumothorax.

**Figure 5:**
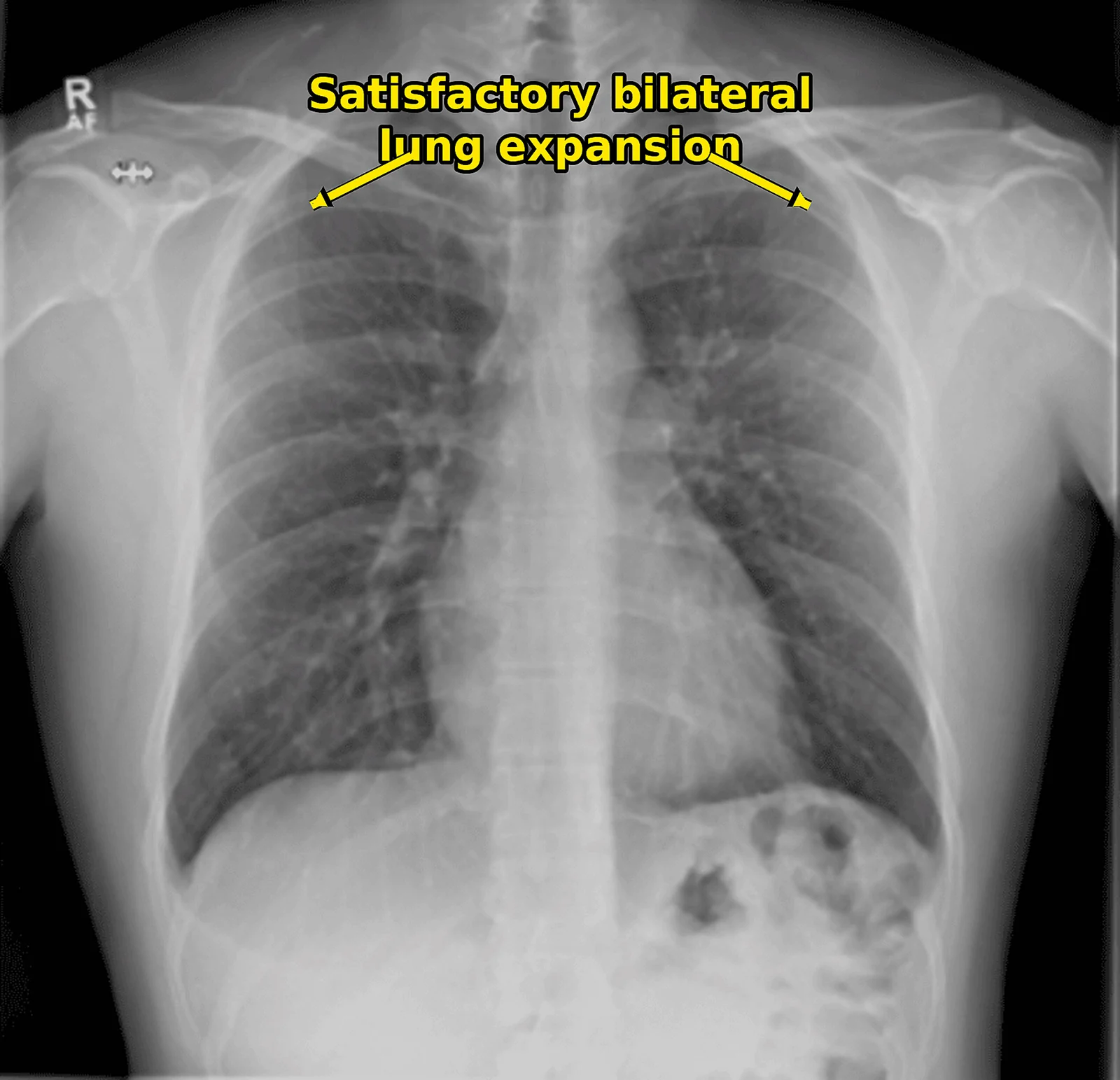
Follow-up chest radiograph obtained eight hours after drain removal: satisfactory bilateral lung expansion without immediate radiographic recurrence

A chest radiograph performed eight hours after drain removal demonstrated satisfactory bilateral lung expansion without immediate radiographic recurrence (Figure 5). The patient was discharged with pleural clinic follow-up, smoking cessation advice, air travel restriction counselling, and advice regarding the risks associated with future thoracic acupuncture.

**Figure 6:**
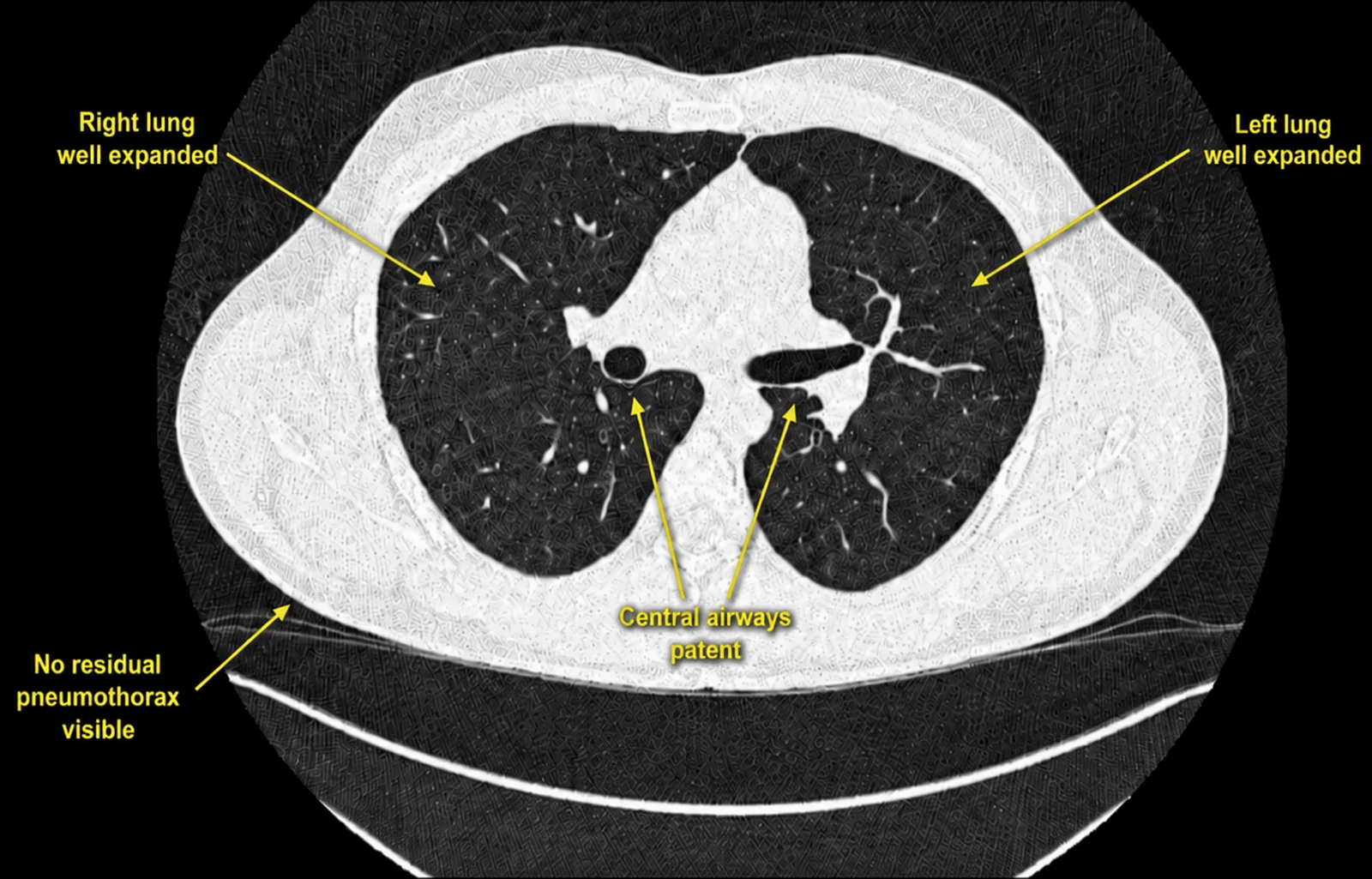
Follow-up CT of the thorax: complete resolution of the previously seen small bilateral pneumothoraces; both lungs are well expanded, with no evidence of underlying bullous or cystic lung disease, focal lung mass, lymphadenopathy, pleural effusion, or parenchymal destruction CT: computed tomography

At pleural clinic follow-up, the patient remained clinically well. His pulse was 68 beats/minute, blood pressure was 110/75 mmHg, and oxygen saturation was 98% on room air. Follow-up CT of the thorax confirmed complete resolution of the previously demonstrated bilateral pneumothoraces, with both lungs fully expanded. There was no evidence of underlying bullous or cystic lung disease, lung mass, lymphadenopathy, pleural effusion, or parenchymal destruction (Figure 6).

The follow-up CT findings were discussed with the patient. He was counselled regarding caution with future thoracic acupuncture and was subsequently discharged from the pleural clinic, with no further follow-up arranged.

Whether the acupuncture practitioner was informed of the complication, whether a formal adverse event report was submitted, and whether pneumothorax had been discussed as part of the pre-procedure consent process were not documented and could not be established retrospectively.

Table 1 provides the timeline of clinical events.

**Table 1:** Clinical timeline CT: computed tomography

Time	Clinical event
Immediately after acupuncture	Sudden onset of pleuritic chest pain, chest tightness, and dyspnoea
Overnight	Symptoms persisted without improvement
Following morning	Presented to the emergency department
Shortly after presentation	Chest radiograph demonstrated a large left pneumothorax with near-complete lung collapse and subtle mediastinal shift
Same day	Left 20 Fr intercostal chest drain inserted
After drain insertion	Repeat chest radiograph demonstrated left lung re-expansion and a small right apical pneumothorax
Same admission	CT of the thorax confirmed left-sided resolution, a small residual right pneumothorax, and no significant structural lung abnormality
Day 3	Chest drain removed following clinical and radiographic improvement
Eight hours later	Chest radiograph demonstrated satisfactory bilateral lung expansion without immediate recurrence
Discharge	Pleural clinic follow-up, smoking cessation advice, air travel restrictions, and counselling regarding future thoracic acupuncture provided
Pleural clinic follow-up	Clinically well; pulse: 68 beats/minute, blood pressure: 110/75 mmHg, and oxygen saturation: 98% on room air
Follow-up CT	Complete resolution of both pneumothoraces with fully expanded lungs and no underlying bullous or cystic lung disease
Following review	CT findings discussed with the patient; discharged from the pleural clinic with no further follow-up arranged

## Discussion

This case is notable because it combines three important features: immediate symptom onset after thoracic acupuncture, delayed hospital presentation, and sequential recognition of bilateral pleural injury. The close temporal and anatomical relationship strongly supports direct pleural puncture as the mechanism of injury. The larger left-sided pleural defect probably produced a more sustained air leak, allowing progressive accumulation of intrapleural air and evolving tension physiology. The smaller right-sided pneumothorax may have resulted from a limited puncture that sealed spontaneously, remaining clinically less apparent until the dominant left-sided pathology had been treated.

The anatomy of the upper back explains this risk. In the cervicothoracic region, the apex of the lung extends above the clavicle, and the posterior pleural surface may lie only a short distance beneath the skin, fascia, and muscle. In the trapezius and infrascapular regions, even modest insertion depths may be hazardous if the needle is directed towards the thoracic cage, particularly in individuals with reduced soft tissue cover [3,4].

Pleural injury may occur when thoracic acupuncture is performed with excessive depth or inappropriate needle direction and may not always be witnessed or recognised immediately during the procedure.

The patient’s BMI was 22.8 kg/m², within the normal range, illustrating that pleural injury may also occur in patients who are not underweight when needling is performed over anatomically high-risk thoracic sites. Immediate pleuritic pain after thoracic acupuncture should therefore not be normalised as a trivial post-treatment effect; it should prompt urgent assessment and clear safety netting because it may indicate pleural irritation or direct pleural breach.

The preserved oxygenation in this case is an additional teaching point. Pulse oximetry may remain deceptively reassuring in young patients with unilateral lung collapse who have no baseline lung disease. Reliance on oxygen saturation alone may therefore delay diagnosis. Careful history-taking and examination remain central. Reduced left-sided air entry and hyperresonance were the key bedside findings that justified immediate imaging despite preserved oxygenation and blood pressure.

The right-sided pneumothorax, identified only after drainage of the left, adds further clinical relevance. Dominant unilateral pneumothorax can anchor clinical attention so strongly that clinicians fail to consider bilateral injury. However, bilateral thoracic needling creates bilateral risk. Repeat imaging after intervention was diagnostically decisive, revealing the contralateral pneumothorax and informing continued observation, discharge counselling, and follow-up.

Management was appropriately asymmetrical. Needle decompression was not performed because the patient remained haemodynamically stable with satisfactory oxygenation and definitive chest drain insertion was immediately available. The chest drain therefore provided prompt and sustained decompression. The small and clinically stable right-sided pneumothorax was managed conservatively. This approach was guided by symptoms, physiological compromise, and clinical trajectory rather than radiographic size alone [8].

CT of the thorax during admission was useful for confirming left-sided resolution, defining the small residual right pneumothorax, and excluding significant structural lung disease. Although smoking was considered a possible background risk factor for spontaneous pneumothorax and recurrence, the immediate onset following bilateral thoracic needling, the anatomical needling sites, and the bilateral pleural injuries strongly supported a procedure-related mechanism.

Follow-up CT subsequently confirmed complete resolution of both pneumothoraces, with fully expanded lungs and no evidence of underlying bullous or cystic lung disease, lung mass, lymphadenopathy, pleural effusion, or parenchymal destruction. These findings further supported an iatrogenic rather than spontaneous cause.

A further strength of the case is the use of digital drainage monitoring. Conventional underwater seal systems depend on subjective interpretation of bubbling and swinging, which can vary among observers. Digital systems can provide objective information on air leak and intrapleural pressure trends, supporting decisions regarding suction, escalation, and drain removal [9]. In this patient, digital monitoring demonstrated cessation of the air leak and supported drain removal once clinical and radiographic stability had been achieved. However, quantitative readings from the device were unavailable retrospectively.

This case also has wider systems safety implications. Acute clinicians should ask specifically about recent acupuncture and other complementary therapies when evaluating unexplained chest pain or dyspnoea. Practitioners performing thoracic acupuncture should understand surface anatomy, adjust insertion depth and direction according to the anatomical site and patient’s body habitus, and provide explicit advice about post-treatment red flag symptoms.

Whether the practitioner was informed, an adverse event was formally reported, or pneumothorax risk had been discussed before acupuncture was not documented. This highlights the importance of informed consent, post-procedure safety netting, communication with the practitioner, and appropriate reporting of serious acupuncture-related complications.

This case has limitations. Serial heart rate measurements after drainage, quantitative digital drainage values, objective measurements of the right-sided pneumothorax, and exact intervals between all investigations were not consistently available retrospectively. Needle dimensions, insertion depth, practitioner qualifications, post-procedure safety netting, and adverse event reporting were also unavailable. However, longer-term clinical and radiological follow-up was obtained, confirming complete resolution and discharge from the pleural clinic.

## Conclusions

Thoracic acupuncture can cause bilateral pleural injury, even when one side dominates the presentation. This case shows how immediate post-procedural pleuritic pain may be followed by delayed attendance, how tension physiology can develop despite preserved oxygen saturation, and how repeat imaging can reveal occult contralateral pneumothorax after treatment of the more obvious side.

Early recognition, decisive drainage of the dominant pneumothorax, objective digital air-leak assessment, and appropriate conservative management of the smaller pneumothorax together produced an excellent outcome. The practical message is clear: when chest pain or breathlessness begins after acupuncture, clinicians should think of pleural injury promptly and should not assume that apparent unilateral findings exclude bilateral complications.
